# Exploring the Effects of Elevated Serum Uric Acid Levels on Hypertension: A Scoping Review of Hyperuricemia

**DOI:** 10.7759/cureus.43361

**Published:** 2023-08-12

**Authors:** Tyagi J Ubhadiya, Nidhi Dubey, Mihir H Sojitra, Karan Shah, Saumya Joshi, Siddharth Kamal Gandhi, Priyansh Patel

**Affiliations:** 1 Department of Internal Medicine, Civil Hospital Ahmedabad, Ahmedabad, IND; 2 Department of Neurology, Civil Hospital Ahmedabad, Ahmedabad, IND; 3 Department of Internal Medicine, M.P. Shah Government Medical College, Jamnagar, IND; 4 Department of Internal Medicine, Medical College Baroda, Vadodara, IND

**Keywords:** deleterious effects, beneficial effects, therapeutic, hypertension, hyperuricemia, serum uric acid level

## Abstract

Hypertension (HTN) is a global health concern due to its increasing prevalence and association with life-threatening complications. An intriguing area of investigation in HTN research is the relationship between HTN and hyperuricemia. In light of this, we conducted a review to summarize the relevant studies exploring the link between elevated serum uric acid (sUA) concentration and new-onset HTN. Through a comprehensive search of PubMed Central, MEDLINE, and PubMed databases, we identified 20 studies that met our inclusion criteria. The research encompassed various study designs, including cohort studies, cross-sectional studies, reviews, and clinical trials.

Pathologically, the elevated sUA levels activate the renin-angiotensin system and also cause the formation of urate crystals, triggering inflammation in the kidneys. Additionally, direct effects on the endothelium contribute to inflammation, oxidative stress, nitric oxide depletion, and smooth muscle cell proliferation, ultimately leading to atherosclerosis. These diverse mechanisms collectively play a role in the pathogenesis of HTN. Interestingly, lowering sUA has been shown to reverse early-stage HTN dependent on uric acid. However, this effect is not observed in the uric acid-independent second stage of HTN. Various studies have demonstrated an independent and dose-dependent association between sUA levels and the prevalence of HTN across different populations and genders. The review highlights the potential role of uric acid-lowering drugs, like allopurinol, in the prevention and early-stage management of HTN. However, there is scarce research on the efficacy of other uric acid-lowering agents and combination therapies. We believe our review provides compelling evidence of the association between elevated sUA concentration and new-onset HTN. Identifying and managing hyperuricemia can provide a preventive approach to reducing the burden of HTN and its associated complications.

## Introduction and background

The prevalence of hypertension (HTN) was found to be 40% in 2008 among individuals 25 years and older worldwide [1]. The prevalence of HTN is on the rise globally, which is a matter of concern as HTN is the leading cause of many life-threatening complications, such as stroke, heart disease, and chronic kidney disease [2-4]. Hence, primordial prevention or elimination of risk factors for HTN is essential as it can lead to a decrease in long-term complications caused by HTN [1,5]. Hyperuricemia is known to be one of the major risk factors for the development of HTN and numerous studies have endorsed this association [6,7]. Hyperuricemia is a condition characterized by elevated levels of serum uric acid (sUA) levels above 7 mg/dL, which is the end product of purine metabolism. This increase in sUA can be attributed to either excessive production of uric acid or reduced uric acid excretion by the kidneys in human beings [2]. One of the chief causes includes genetic polymorphisms in anion transporters such as URAT1/SLC22A12, GLUT9/SLC2A9, and ABCG2/BCRP [2,7], among which defect in ABCG2 exporter is the prime cause of hyperuricemia and gout by decreasing renal urate excretion [2]. Lifestyle and diet preferences such as a diet rich in fats, meats, seafood, fructose, and alcohol can be important causes of the explosion in hyperuricemia incidence. Excessive cell death or cell turnover can also cause hyperuricemia due to an increase in serum purine [7]. Uric acid may increase blood pressure (BP) through oxidative stress, nitric oxide reduction, renin-angiotensin system activation, endothelial inflammation, vascular smooth muscle proliferation, and insulin resistance [4,6]. In this review article, we summarize the relevant studies that show the association between elevated sUA concentration and new-onset HTN.

## Review

Methodology

We conducted a thorough search of PubMed Central, MEDLINE, and PubMed databases. The following search strategy was selected based on the medical subject headings regular search: Uric Acid AND Hypertension, 2070 articles were found, and then articles were searched by using (MeSH) vocabulary: (((( "Uric Acid/adverse effects"[Majr] OR "Uric Acid/metabolism"[Majr] OR "Uric Acid/toxicity"[Majr] )) AND (( "Hypertension/classification"[Majr] OR "Hypertension/enzymology"[Majr] OR "Hypertension/epidemiology"[Majr] OR "Hypertension/etiology"[Majr] OR "Hypertension/immunology"[Majr] OR "Hypertension/metabolism"[Majr] OR "Hypertension/pathology"[Majr] OR "Hypertension/physiopathology"[Majr] ))) NOT ("Diabetes Mellitus, Type 2"[Majr])) NOT Kidney Disease NOT pregnancy NOT preeclampsia. No time limits were imposed, and all types of studies in the literature published in English with full text were included in the selection process. However, articles were excluded if their free full text was not retrievable, and duplicate publications were also excluded from the final selection. All articles were screened and disagreements were discussed among all the authors until consensus was achieved. After discussion among all the authors, a total of 20 studies were finalized to be included in the review. The research includes six cohort studies, seven cross-sectional studies, four review articles, one systematic review and meta-analysis, one case-control study, and one clinical trial.

Pathophysiology

Uric acid can contribute to the development of HTN through several mechanisms, including indirect effects on the kidneys and direct effects on the endothelium.

Effect on Kidneys

Hyperuricemia can lead to the formation of urate crystals in the kidneys, causing inflammation and injury to the renal tissues and also by activation of the intrarenal renin-angiotensin system [8-10].

Effect on Endothelium

**Figure 1:**
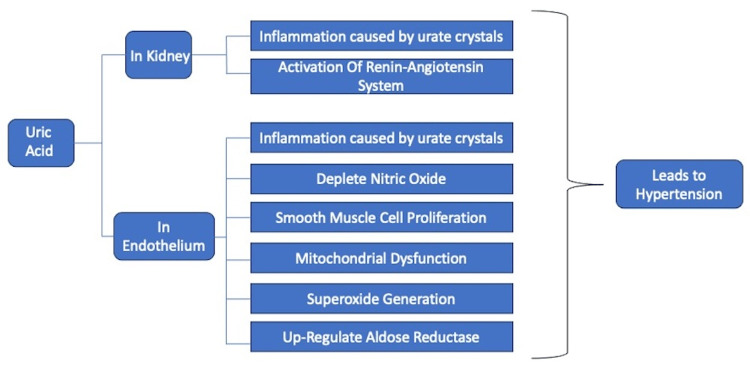
Pathophysiology of the development of hypertension Image credits: Tyagi Ubhadiya, Priyansh Patel, Nidhi Dubey

Elevated levels of sUA can impair endothelial function, leading to endothelial dysfunction by various mechanisms. Uric acid crystals deposition in endothelial cells of blood vessels causes inflammation leading to hardening of blood vessels [8-10]. Active oxygen is produced in the process of generating uric acid from hypoxanthine by xanthine oxidase. These active oxygen species cause depletion of nitric oxide in endothelium leading to smooth muscle proliferation, thereby promoting atherosclerosis [8-10]. Uric acid itself flows into the cells via a uric acid transporter present in the vascular endothelial cells and vascular smooth muscle cells and causes smooth muscle cell proliferation [8-10]. Uric acid may function as an antioxidant against extracellular-induced oxidative stress, but when uric acid enters cells, it exhibits pro-oxidant properties by causing mitochondrial dysfunction and superoxide generation through the activation of nicotinamide adenine dinucleotide phosphate (NADPH) oxidases, thus depleting adenosine triphosphate (ATP) [9,10]. Soluble uric acid induces an increase in aldose reductase expression within the endothelium and other tissues, leading to the activation of the polyol pathway. This, in turn, has two primary outcomes: inhibition of nitric oxide production and the generation of endogenous fructose [9]. Figure 1 shows the pathophysiology of the development of HTN in association with raised sUA.

**Figure 2:**
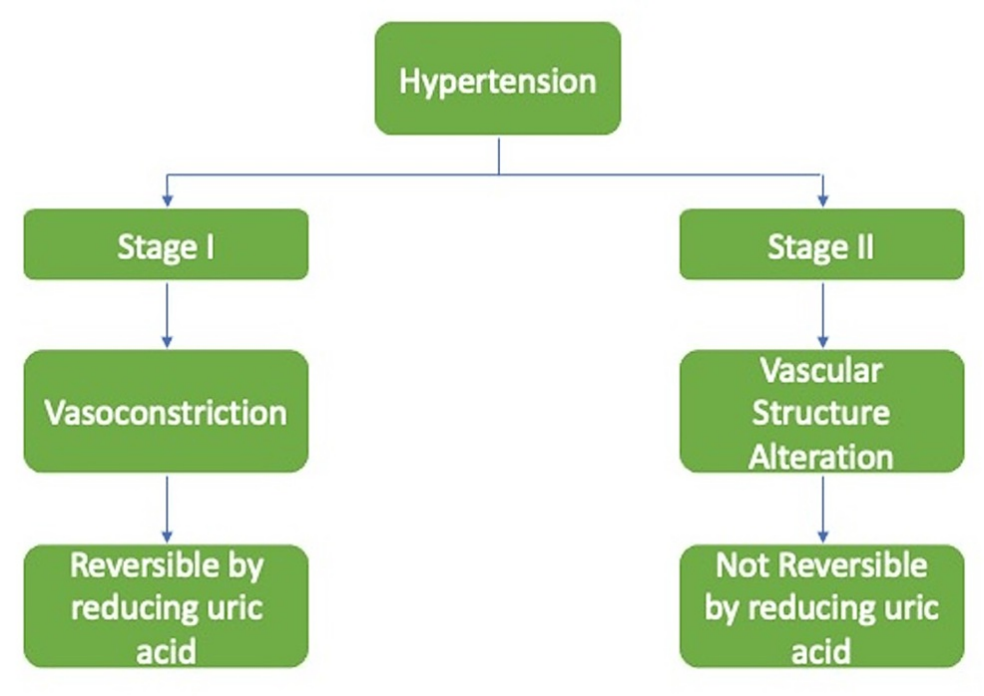
Stages of hypertension Image credits: Tyagi Ubhadiya, Priyansh Patel, Nidhi Dubey

HTN caused by elevated uric acid unfolds in two stages. In the first stage, there is significant vasoconstriction due to the inhibition of nitric oxide synthesis and the activation of the renin-angiotensin system. Lowering sUA levels can reverse HTN during this stage since it is dependent on uric acid. However, in the second stage, alterations in the vascular structure occur as a result of the proliferation of vascular smooth muscle cells, ultimately leading to an elevation in BP. This phase is uric acid-independent and there is no effect of lowering sUA on HTN [8]. Figure 2 shows the stages of HTN in association with raised sUA.

Complications

Hyperuricemia is widely acknowledged as a significant risk factor for the onset of HTN. Numerous studies have investigated the correlation between sUA levels and the occurrence of HTN. One such study by Wei et al. in a Chinese population comprised a cross-sectional study at baseline, as well as a cohort study that followed subjects without HTN for a period of six years, considering sUA levels as a dynamic component. The results of this study indicated that hyperuricemia is independently linked to the occurrence of HTN, and the connection between these two factors is dose-dependent [11]. Sung et al. examined non-hypertensive Korean subjects and followed them up for eight years in a cohort study. Data were collected at baseline, as well as first and second visits; 10,405 out of 96606 subjects developed HTN. At the follow-up, it was observed that subjects who developed HTN had higher average sUA concentration and the risk of incident of HTN increased progressively across the quartiles of uric acid in both men and women. It was also observed that high initial sUA concentration and increases in sUA between the first and second visits in particular were associated with an increased risk of incident HTN. The hazard ratio (HR) for incident HTN, after adjusting for confounding variables, was found to be 1.29 in men and 1.24 in women when comparing the highest versus the lowest quartiles of uric acid levels. Even after excluding those who were on anti-hypertensive medication at follow-up and including subjects with a history of coronary artery disease, cardiovascular disease, cancer, diabetes, and metabolic syndrome, the results did not change [12]. In a cohort study of Japanese males aged 18-60 years, 43% developed HTN after a mean follow-up of 7.2 years. sUA was divided into three tertiles. Incidence of HTN increased with an increase in mean sUA, i.e., 37.4% in the lowest tertile and 50.8% in the highest tertile. The HR for incident HTN remained significant before and after adjusting the confounding variables but HR was comparatively higher in participants aged more than or equal to 40 years than those aged less than 40 years [8].

In a cross-sectional study conducted in China, participants were categorized into three age groups: 41-50 years, 51-60 years, and 61-70 years. The findings revealed that the prevalence of HTN and hyperuricemia was notably higher among individuals in the age group of 61-70 years. However, the association between hyperuricemia and HTN was found to be significant only in the age group of 41-50 years [3]. The systemic review and meta-analysis conducted by Wang et al. revealed that elevated uric acid precedes the development of HTN and is linked to HTN in a continuous and dose-dependent manner. Furthermore, the association between sUA and the development of HTN was found to be independent of other risk factors for HTN [13]. In a prospective observational study by Chen et al., in which subjects were followed up for five years, 6.55% were diagnosed with HTN. Subjects who developed HTN at the end of the study had higher baseline sUA and SBP and DBP. The study's findings indicated that elevated sUA levels were strongly linked to a higher incidence of newly developed HTN. Moreover, the incidence of HTN exhibited a sudden increase when sUA levels exceeded 500 mmol/L, suggesting a critical threshold effect. Regarding the interaction between sUA and other independent risk factors in the development of new HTN, triglyceride levels demonstrated a significant interaction. However, variables such as sex, age, body mass index (BMI), fasting plasma glucose (FPG), and high-density lipoprotein (HDL) cholesterol did not show a significant interaction with sUA in terms of HTN development [14]. A cross-sectional study by Ouppatham et al. yielded similar results: a positive correlation between higher sUA levels and elevated SBP and DBP was seen in the Thai army population [15].

Kansui et al. conducted a cross-sectional study of males ranging in age from 18 to 64 years, where subjects were divided into six groups, according to the classification of BP in adults based on The Japanese Society of Hypertension Guidelines for the Management of Hypertension (JSH 2009), into optimal BP, normal BP, high-normal BP, grade I HTN, grade II HTN, and grade III HTN. In all subjects, sUA was significantly elevated in a linear fashion as BP increased especially in subjects with grade II and III HTN. Even after the exclusion of the subjects who received anti-hypertensive or uric acid-lowering drugs, the association remained the same; however, when the subjects with anti-hypertensive drugs, but without uric acid-lowering drugs, were studied, the association of sUA with the level of BP was not found [16]. In a study involving 1496 women aged 32-52 years, it was discovered that for each 1 mg/dL rise in uric acid, there was a 1.33 times higher likelihood of developing HTN after controlling for matching factors, and multivariable adjustment for BMI, physical activity, smoking, alcohol intake, and family history of HTN. The odds ratio (OR) for the highest quartile compared to the lowest quartile was found to be 2.17. Furthermore, after additional adjustments for estimated glomerular filtration rate (eGFR), levels of total cholesterol, triglycerides, insulin, homocysteine, and soluble intercellular adhesion molecule-1 (sICAM-1), it was found that every 1 mg/dL increase in uric acid level was associated with a 1.25-fold increase in odds of incident HTN. The OR in the highest quartile when compared with the lowest quartile of uric acid level was 1.89 [17].

In a cross-sectional study among the Nepalese population, hyperuricemia was found in 28.4% of male hypertensive, 29.2% of female hypertensive, and 28.8% of total hypertensive cases. Similarly, hyperuricemia was found in 14.3% of male healthy controls, 12.9% of female healthy controls, and 13.7% of total healthy controls. After adjusting for BMI, a moderate decrease in the correlation between the sUA level and HTN was observed [18]. Many studies have been performed to examine the effect of uric acid on various genders, and different studies have come to different results. Ali et al. conducted a cross-sectional study that concluded that both SBP and DBP increased with elevated concentrations of sUA in the quartiles in both genders. However, a stronger relationship between sUA concentration with HTN and pre-HTN was observed in females compared to male participants [2]. Additional studies have offered further evidence supporting the correlation between elevated sUA levels and the prevalence of HTN in both genders. In a cross-sectional study, after adjusting for various factors such as age, BMI, dyslipidemia, diabetes mellitus, alcohol consumption, smoking, and eGFR, it was found that sUA levels more than or equal to 5.3 mg/dL in men and more than or equal to 4.3 mg/dL in women were significantly associated with HTN. Hypertensive men were 1.79 times more likely to have hyperuricemia compared to normotensive men, while hypertensive women were nearly six times more likely to have hyperuricemia compared to normotensive women [4]. One potential reason for the greater impact of sUA on BP and cardiovascular mortality in women compared to men could be the presence of lower levels of sUA in women. Hence, the effects of elevated sUA levels might be more pronounced in women [16]. A study conducted by Yu et al. revealed a higher prevalence of HTN and hyperuricemia in males compared to females. The study suggests that the stronger association between hyperuricemia and the risk of HTN in males could be attributed to the fact that estrogen, present in females, acts as a uricosuric agent, thereby providing a protective effect against hyperuricemia [19].

Prevention and treatment

In the early stages of HTN, it has been observed that lowering uric acid levels through lifestyle modification with dietary changes or through the use of uric acid-lowering agents can provide a protective effect. By lowering uric acid, these drugs may help in mitigating the risk factors associated with HTN and potentially slow down its progression [13]. In the meta-analysis conducted by Wang et al., a randomized, double-blind, placebo-controlled, cross-over trial was mentioned. The trial focused on 30 adolescents who had recently been diagnosed with stage 1 essential HTN and hyperuricemia and had not received any prior treatment for HTN. They were treated with allopurinol, a uric acid-lowering drug, as well as a placebo, with an appropriate washout period between the two treatments. While receiving allopurinol treatment, 20 out of the 30 participants attained normal BP, whereas only one out of 30 participants achieved normal BP during the placebo treatment. This suggests that allopurinol treatment demonstrated greater effectiveness in achieving normal BP compared to the placebo [13]. In an open-label crossover study involving five children with newly diagnosed and untreated essential HTN, the subjects received allopurinol treatment for four weeks, followed by a six-week washout period, allowing the medication to be eliminated from their systems. After the treatment, four out of the five children experienced a decrease in BP. However, six weeks post-treatment cessation, the BP levels of all five children reverted to their initial baseline levels [20].

Limitations

This study has a few limitations that should be acknowledged. Firstly, the coverage of literature was limited to three selected databases, namely PubMed Central, MEDLINE, and PubMed. Consequently, only free full-text articles were included, which may have excluded potentially relevant information. In addition, the majority of studies analyzed primarily focused on the East Asian population, making it challenging to generalize the findings to other populations. Moreover, the review only included papers published in English, thereby excluding studies published in other languages. Furthermore, there was a scarcity of studies investigating the effects of uric acid-lowering drugs on hypertension.

## Conclusions

Our findings indicate a significant relationship between higher uric acid levels and the incidence of HTN, emphasizing the importance of considering uric acid as a potential risk factor in the development of this condition. High sUA can lead to HTN through various mechanisms, including inflammation in the kidney and endothelium, activation of the renin-angiotensin system, depletion of nitric oxide, smooth muscle cell proliferation, superoxide generation, mitochondrial dysfunction, and activation of polyol pathway. A direct and dose-dependent association of uric acid level on increased SBP and DBP in both males and females has been established. Early detection of conditions associated with high uric acid levels, such as extremely rare genetic diseases or conditions influenced by diet, lifestyle modifications (including dietary changes), and the appropriate use of uric acid-lowering drugs can play a significant role in managing HTN and minimizing the potential complications associated with high uric acid levels. Further large-scale multicenter studies involving oral medications that lower uric acids, beyond allopurinol, such as other agents or combination therapies, could provide a broader perspective on their impact on HTN management.
